# Home monitoring vs hospitalization for mild acute pancreatitis. A pilot randomized controlled clinical trials

**DOI:** 10.1097/MD.0000000000033853

**Published:** 2023-05-17

**Authors:** Maria Sorribas, Thiago Carnaval, Núria Peláez, Luis Secanella, Silvia Salord, Sònia Sarret, Sebastián Videla, Juli Busquets

**Affiliations:** a Digestive and General Surgery Department, Bellvitge University Hospital, L’Hospitalet DE Llobregat, Barcelona, Spain; b Research Group of Hepato-Biliary and Pancreatic Diseases, Institut d’Investigació Biomèdica de Bellvitge – IDIBELL, University of Barcelona, L’Hospitalet DE Llobregat, Barcelona, Spain; c Pharmacology Unit, Department of Pathology and Experimental Therapeutics, School of Medicine and Health Sciences, IDIBELL, University of Barcelona, L’Hospitalet DE Llobregat, Barcelona, Spain; d Gastroenterology Department, Bellvitge University Hospital, L’Hospitalet DE Llobregat, Barcelona, Spain; e Home Hospitalization Unit, Bellvitge University Hospital, L’Hospitalet DE Llobregat, Barcelona, Spain; f Clinical Research Support Unit (HUB·IDIBELL), Clinical Pharmacology Department, Bellvitge University Hospital, L´Hospitalet DE Llobregat, Barcelona, Spain; g Departament de Ciències Clíniques, Facultat de Medicina i Ciències de la Salut, Universitat de Barcelona (UB), Barcelona, Spain.

**Keywords:** acute pancreatitis, early oral feeding, home monitoring

## Abstract

Acute pancreatitis is a high-incidence benign disease. In 2009, it was the second highest cause of total hospital stays, the largest contributor to aggregate costs (approximately US$ 7000.00 per hospitalization), and the fifth leading cause of in-hospital deaths in the United States. Although almost 80% of acute pancreatitis cases are mild (usually requiring short-term hospitalization and without further complications), severe cases can be quite challenging.

Classifications, scores, and radiological criteria have been developed to predict disease severity and outcome accurately; however, in-hospital care remains of widespread use, regardless of disease severity. A recent Turkish study reported that mild acute pancreatitis can be effectively and safely managed with home monitoring. Although the optimal timing for oral refeeding remains controversial and could cast some doubt on the feasibility of home monitoring, some guidelines already advocate for starting it within 24 hours.

The present clinical trial aims to assess whether home monitoring is effective, safe and non-inferior to hospitalization for managing mild acute pancreatitis.

**Methods:**

This will be a multicenter open-label randomized (1:1) controlled clinical trial to assess the efficacy and safety of home monitoring compared to in-hospital care for mild acute pancreatitis. All patients coming to the emergency department with suspected acute pancreatitis will be screened for enrollment. The main variable will be treatment failure (Yes/No) within the first 7 days after randomization.

**Discussion:**

Acute pancreatitis implies a high economic burden in healthcare systems worldwide. Recent evidence suggests that mild disease can be safely and effectively treated with home monitoring. This approach may produce considerable cost savings and positively impact patients’ quality of life. We expect the results to show that home monitoring is effective and not inferior to hospitalization for managing mild acute pancreatitis and that the economic costs are lower, kickstarting similar trials throughout the world, optimizing the use of limited healthcare budgets, and improving patients’ quality of life.

## 1. Introduction

### 1.1. Background

Acute pancreatitis (AP) is a high-incidence benign disease. In the United States, the estimated annual incidence ranges from 110 to 140 cases/100,000 people^[1]^ and is the most frequent principal diagnosis at discharge in gastrointestinal disease and hepatology.^[2]^ AP admissions have increased from 9.48 cases/1000 hospitalizations in 2002 to 12.19 in 2013, and the median hospital cost is estimated at approximately US$ 7000.00 per hospitalization.^[1]^ In 2009, AP was the second highest cause of total hospital stays, the largest contributor to aggregate costs, and the fifth leading cause of in-hospital deaths. Similarly, the annual incidence in Spain reaches 72 cases/100,000 people per year.^[3]^

Severe AP can be quite challenging, usually requiring long-term hospitalizations, intensive care, and/or surgical treatment. Fortunately, almost 80% of APs are mild, usually requiring short-term hospitalization (approximately 5 days) without further complications. Different trustworthy criteria, such as the Atlanta classification, Ranson score, and the Bedside Index for Severity in Acute Pancreatitis (BISAP) score, have been developed to predict disease severity and outcome.^[1]^ Notwithstanding, in-hospital care remains of widespread use, regardless of disease severity.

Recently, a Turkish pilot study reported that patients with mild nonalcoholic AP could be safely monitored at home when undergoing regular visits by a nurse under physician oversight.^[4]^ All patients were monitored as inpatients for up to 24 hours from admission; they received intravenous fluid therapy until oral feeding resumption. Although the optimal timing for oral refeeding remains controversial^[5]^ and could cast some doubt on the feasibility of home monitoring, different guidelines recommend its early start in AP: the International Association of Pancreatology and the American Pancreatic Association advocate starting once abdominal pain is decreasing and inflammatory markers are improving^[1,6]^; the American Gastroenterological Association recommends starting within 24 hours; and the American College of Gastroenterology recommends starting it within 48 to 72 hours unless not tolerated or contraindicated.^[1]^ Additionally, some authors recently reported that immediate oral feeding is feasible, safe and may accelerate recovery.^[5,7–9]^

Accordingly, we designed the present clinical trial to assess whether home monitoring is effective, safe and non-inferior to hospitalization for managing mild AP.

### 1.2. Working hypotheses and objectives

The current study protocol has 2 working hypotheses:

1. home monitoring is effective and safe for managing mild AP.
2. home monitoring is not inferior to hospitalization for managing mild AP.

Our main objective is to assess the efficacy of home monitoring compared to in-hospital care for mild AP.

Our secondary objectives are:

• To assess the safety of home monitoring by estimating the incidence of AP-related complications during the first 30 days after diagnosis.
• To study the readmission rate during the first 30 days after diagnosis.
• To estimate the incidence of mortality at 30 days.
• To assess the quality of life (QoL).
• To study treatment-related costs.

## 2. Methods

### 2.1. Study design

**Figure 1.**
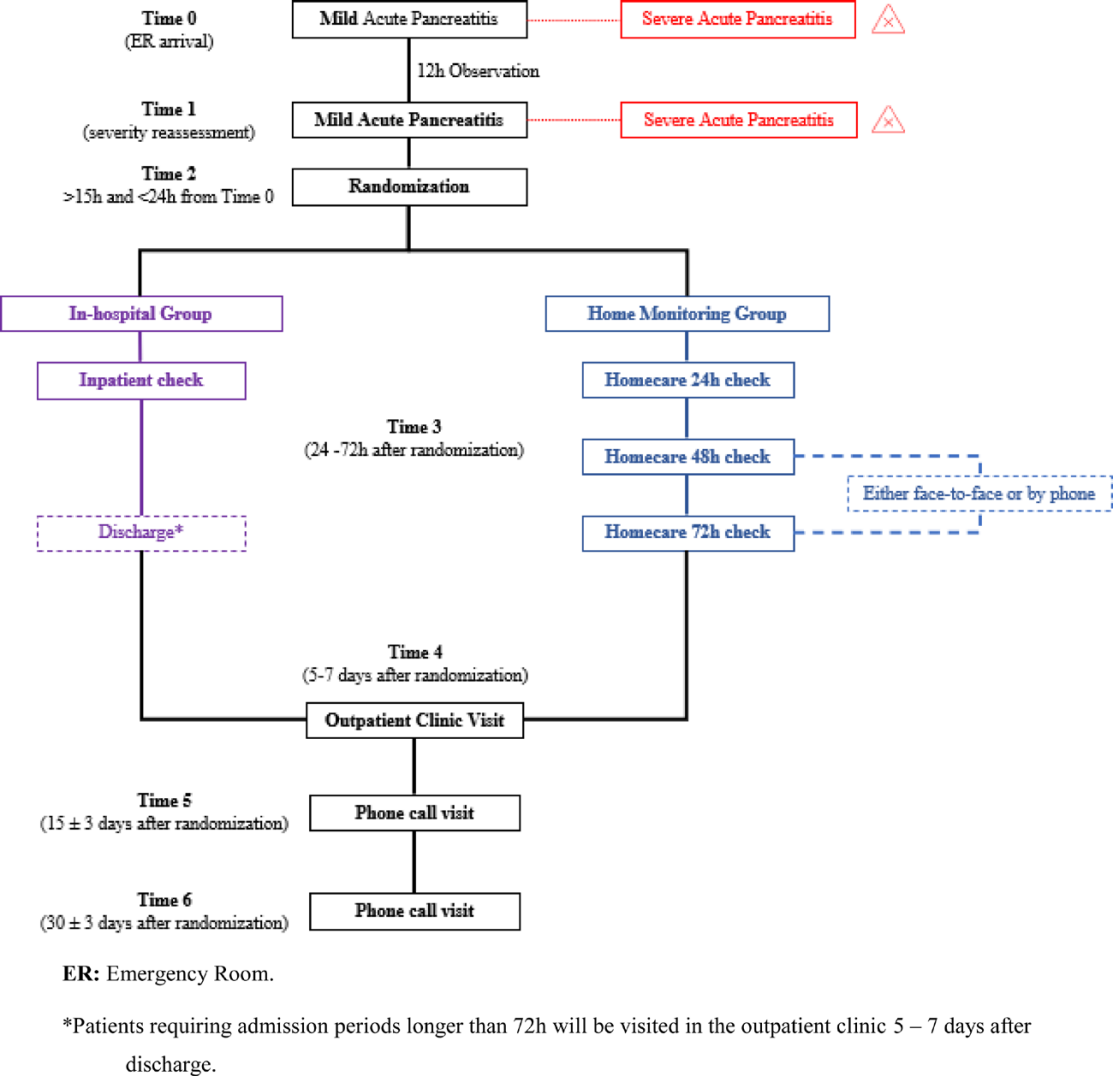
Study Flowchart. ER = emergency room. *Patients requiring admission periods longer than 72 hours will be visited in the outpatient clinic 5–7 days after discharge.

This will be a multicenter open-label pilot randomized controlled clinical trial to assess the safety, efficacy, and non-inferiority of home monitoring compared to in-hospital care for mild AP. Patients will be randomly assigned (1:1) to either the home monitoring (experimental) group or the in-hospital (control) group (see Fig. 1).

All patients with abdominal pain and clinical suspicion of acute pancreatitis will be initially evaluated by each center’s emergency room (ER) specialist physician. Once the diagnosis of mild AP is confirmed (Time 0), we will follow the 2012 Revised Atlanta Classification Criteria^[10]^ to assess disease severity. Supportive care will be provided, and patients will be reassessed for severity 12 hours after the first assessment (Time 1) (see Fig. 1).

Supportive care in the ER at Time 0 will consist of intravenous administration of fluid therapy, analgesia with acetaminophen 1 g/8 h interchanged with dipyrone 575 mg/8 h (or Dexketoprofen 25 mg/8 h, if allergies/intolerance); antiemetic treatment with Ondansetron 4 mg/8 h; gastroprotective treatment with Omeprazole 20 mg/24 h. After 12 hours in the ER (Time 1), patients will be reassessed for severity and, if adequate pain control (Visual Analogue Scale [VAS] ≤ 3) and no nausea or vomits, we will assess oral feeding tolerance.

After 15 hours and before 24 hours after Time 0 (Time 2), patients still presenting VAS ≤ 3 and adequate oral feeding tolerance will be fully informed about the study, provided with the information sheet and invited to participate. If they accept, we will ask them to sign the written informed consent. Afterward, we will perform the randomization, and patients will be informed of their assigned study group.

### 2.2. Settings

This multicenter study will be carried out at the following medical centers:

- Hospital Univeritari de Bellvitge;
- Consorci Corporació Sanitària Parc Taulí de Sabadell;
- Hospital Sant Joan Despí Moises Broggi—Consorci Sanitari Integral;
- Hospital de Viladecans;
- Hospital Hospital de Igualada - Consorci Sanitari de l’Anoia;
- Hospital Fundació Sant Joan de Déu de Martorell;
- Hospital Germans Trias i Pujol de Badalona;
- Hospital de Terrassa;
- Hospital Universitari Vall d’Hebron;
- Hospital de la Santa Creu i Sant Pau.

### 2.3. Eligibility criteria

Patients who meet all inclusion criteria and none of the exclusion criteria will be included in the RHINO-trial.

#### 2.3.1. Inclusion criteria.

• Adult patients (≥18 and ≤80 years of age);
• Both sexes;
• Diagnosed with mild AP by at least 2 of the following 3 criteria:
  1. Abdominal pain;
  2. Plasma/urine amylase or lipase levels ≥ 3× upper limit of normal;
  3. Imaging tests (Abdominal ultrasound/ computerized tomography scan) showing signs of AP.
• Lack of potential severity criteria (at randomization), i.e., presenting none of the following:
  1. Systemic inflammatory response syndrome^[11]^;
  2. Plasma C-reactive Protein levels ≥ 150 mg/dL;
  3. Marked increase in the White Blood Cell Count;
  4. Coagulopathy (INR > 1.4);
  5. Hematocrit > 44%;
  6. Blood Creatinine > 170 µmol/L;
  7. BISAP score > 2.
• Satisfactory pain control (VAS ≤ 3) after 12 hours of treatment in the ER;
• Satisfactory oral feeding tolerance (i.e., no repeated vomiting episodes);
• Lack of evidence of AP-related complications (systemic or local) in the imaging tests;
• Adequate cognitive capacity to follow medical orders;
• Patients meeting each center’s logistic homecare criteria;
• Patients who give their written informed consent.

#### 2.3.2. Exclusion criteria.

• Past medical history of pancreatic disease such as:
  1. Known or *de novo* chronic pancreatitis (e.g., Wirsung duct dilation, pancreatic calcifications).
  2. Recurrent AP (>3 flare-ups/year).
  3. Previous AP flare-up within 1 month.
  4. AP after endoscopic retrograde cholangiopancreatography.
• Hyperbilirubinemia > 3× upper limit of normal.
• Past medical history of acute myocardial infarction, cirrhosis, chronic kidney disease, and/or chronic pulmonary disease.
• Body mass index ≥ 35 kg/m^2^.
• Patients who refuse to participate in the trial.

### 2.4. Interventions and criteria for discontinuing allocated interventions

#### 2.4.1. Home monitoring group.

Patients randomized to this group will be discharged and monitored through homecare. They will be prescribed an easy-digestion diet plan and oral analgesics. A face-to-face visit will be performed for all patients 24 hours after randomization (Time 3) by both a nurse and a physician from the Homecare Department. If they deem necessary, face-to-face visits will be performed again 48 and 72 hours after randomization. Otherwise, these visits will be conducted through a phone call.

Patients will be handed the *Participant Diary* after hospital discharge where they will register their maximum daily pain intensity, daily VAS score (at 8 pm), and daily oral feeding tolerance until the outpatient clinic visit (Time 4).

Should the homecare team suspect any complications, patients will be referred to the hospital for a full evaluation. The last face-to-face study visit (Time 4) will be performed at the outpatient clinic for all participants 5 to 7 days after randomization. During this visit, patients will be asked to fulfill the EuroQoL questionnaire for health-related QoL (HRQoL) assessment. A phone call visit will be performed at 15 (± 3) days (Time 5) and 30 (± 3) days (Time 6) for all patients for overall assessment.

#### 2.4.2. In-hospital group.

Patients randomized to this group will be admitted under the care of physicians from the Digestive and General Surgery Department or the Gastroenterology Department. Patients will be visited daily during admission and will be discharged according to their clinical evolution; however, they will remain at in-hospital care for at least 24 hours, as per standard clinical practice. They will initially receive liquid diet and intravenous analgesia; blood tests will be performed at 24 hours after randomization. Oral feeding progression will be performed according to individual tolerance.

Patients will be handed the *Participant Diary* where they will register their maximum daily pain intensity and VAS score daily (at 8 pm) until the outpatient clinic visit (Time 4) – last face-to-face study visit. During this visit, patients will be asked to fulfill the EuroQoL questionnaire for HRQoL assessment and to deliver the *Participant Diary*. A phone call visit will be performed at 15 (± 3) days (Time 5) and 30 (± 3) days (Time 6) for all patients for overall assessment. Notably, patients requiring admission periods longer than 72 hours will be visited in the outpatient clinic 5 to 7 days after discharge. Accordingly, phone call visits will be performed at 15 (±3) days and 30 (±3) days after discharge.

After the Outpatient Clinic visit, all patients diagnosed with a lithiasic pancreatitis (regardless of their randomized study group) will undergo evaluation for scheduling a cholecystectomy.

#### 2.4.3. Criteria for discontinuing allocated interventions.

Patients allocated to the home monitoring group will be discontinued if their clinical status worsens and they require further admission. We will also discontinue patients who request to be withdrawn from the study and those who fail to comply with study-related procedures.

### 2.5. Outcomes

Our primary outcome measure will be the 7-day (after randomization) treatment failure rate. Treatment failure is defined as a VAS > 3 and/or oral feeding intolerance (nausea, repeated vomiting episodes, early satiety).

Our secondary outcome measures will be:

• The cumulative incidence of complications* secondary to AP during the first 30 days after diagnosis.

*Note: AP-related complications include, but are not limited to, abscess formation, pseudocysts, local necrosis, kidney failure, respiratory failure.

• The number (percentage) of patients readmitted to the hospital during the first 30 days after diagnosis.
• The cumulative incidence of mortality during the first 30 days (30-day mortality) after diagnosis.
• The median (95% CI) Charlson Comorbidity Score per group.
• The median (95% CI) EuroQoL questionnaire score.
• The estimated costs (95% CI) of each intervention.

### 2.6. Participant timeline

Participant timeline is summarized in Table 1

**Table 1:** Participant timeline.

	ER						
	Time 0	Time 1	Time 2	Time 3	Time 4	Time 5	Time 6
	ER arrival	≤12 h from Time 0	>15 h and <24 h from Time 0	24-72 h after Rd	5–7 d after Rd	15 d (±3) after Rd	30 d (±3) after Rd
Diagnostic assessment	**✓**	**✓**					
VAS assessment	**✓**	**✓**	**✓**				
Complementary tests							
Blood tests*	**✓**			**✓**			
Abdominal and thoracic plain radiography	**✓**						
Abdominal ultrasound or CT scan	**✓**	**✓**					
Oral feeding:							
Water/jellified water		**✓**	**✓**				
IV Fluid Therapy				**✓**			
Tolerance assessment				**✓**	**✓**		
Easy digestion oral feeding				**✓**			
Oral feeding progression				**✓** †			
Supportive care‡	**✓**	**✓**	**✓**	**✓**			
Hospital discharge			**✓**	**✓** §			
Homecare assessment				**✓**			
VAS Assessment				**✓**	**✓**		
Outpatient Clinic Assessment					**✓**		
EuroQoL questionnaire					**✓**		
Phone call visit						**✓**	**✓**
Written informed consent			**✓**				
Randomization			**✓**				
Adverse events			**✓**	**✓**	**✓**	**✓**	**✓**

CT = computerized tomography, IV = intravenous, ER = emergency room, Rd. = randomization, VAS = Visual Analogue Scale.

**✓**: All patients; **✓**: Home Monitoring Group patients only; **✓**: In-Hospital Group patients only.

* *Blood tests*: Plasma amylase and/or lipase, arterial or venous blood gas testing (for pH assessment), Complete blood count, urea, creatinine, sodium, potassium, C reactive Protein, alanine aminotransferase, aspartate aminotransferase, bilirubin, Prothrombin Time, activated Partial Thromboplastin Time.

† *Oral feeding progression: Home Monitoring Group*: Low-fat and low-residue diet consisting of a daily 1800 kcal intake (110 g of proteins); *In-Hospital Group*: Liquid diet.

‡ *Supportive Care: ER*: IV analgesia for all participants; *Home Monitoring Group*: Oral analgesia; *In-Hospital Group*: Intravenous analgesia (during at least the first 24 hours of admission) followed by (2) Oral analgesia once tolerated.

*IV analgesia*: Acetaminophen 1 g/8 h alternated with dipyrone 575 mg/8 h (or Dexketoprofen 25 mg/8 h, if allergies/intolerance), plus Ondansetron 4 mg/8 h, plus Omeprazole 20 mg/24 h.

*Oral analgesia*: Acetaminophen 1 g/8 h alternated with dipyrone 575 mg/8 h (or Dexketoprofen 25 mg/8 h, if allergies/intolerance), plus Omeprazole 20 mg/24 h.

§ Patients requiring admission periods longer than 72 hours will be visited in the outpatient clinic 5–7 days after discharge. Accordingly, phone call visits will be performed at 15 (±3) days and 30 (±3) days after discharge.

### 2.7. Sample size

Previously published data^[12]^ reported therapeutic success rates (absence of rescue analgesia and proper oral feeding tolerance) during the first 7 days after diagnosis is approximately 95%. Considering a non-inferiority margin of 10%, an expected success rate in the experimental group greater than 90%, an 80%-statistical power, and a 2.5% type-I error for a one-sided hypothesis, we estimated that 202 patients would be required to reject the null hypotheses (*H*_*0*_*: home monitoring is not effective and is inferior to hospitalization for managing mild AP*). Taking into account a possible 10% dropout rate (loss to follow-up), we will include 154 patients per study group.

An interim analysis (for safety purposes) will be performed when half the estimated sample size is included. A detailed report of all recorded adverse events will be provided to the Data Safety Monitoring Committee (DSMC).

### 2.8. Recruitment

Patients coming to the ER with suspected AP will be screened for enrollment. A study team member will evaluate them in the ER following the steps detailed in Figure 1 and Table 1. As mentioned before, Time 0, Time 1, and Time 2 will be the same for all screened patients. Once the recruiting investigator ensures that patients meet all the inclusion criteria and none of the exclusion criteria, he/she will explain the trial thoroughly and invite them to participate. If they accept to participate, they will be required to sign the written informed consent. Afterward, randomization will take place.

### 2.9. Allocation (sequence generation, concealment, implementation) and blinding

Patients will be randomly allocated to either the experimental or the control group following a parallel group design and a 1:1 allocation ratio. A total of 308 opaque envelopes (154 for the experimental group and 154 for the control group) will be prepared prior to the study start. The envelopes will be stored in a secure vault and will be randomly opened by the time of the randomization.

Since this is an open-label study, blinding (masking) is not applicable.

### 2.10. Data collection plan

Patients’ data will be gathered through clinical interview, clinical examination, and by reviewing the electronic medical records and the Participant Diary (provided to each participant after randomization).

We will gather information on:

• Baseline characteristics: age, sex, recruiting hospital, body mass index, and the ASA score (American Society of Anesthesiologists’ classification scale of overall physical health).
• AP etiology.
• Symptoms presented and their onset date.
• ER discharge date (Home Monitoring Group).
• Hospital admission and discharge dates (In-Hospital Group).
• BISAP score and Charlson Comorbidity Index at diagnosis.
• Clinical data: Vital signs; VAS score; oral feeding tolerance.
• Laboratory and Imaging data.
• ER attendance and/or hospital readmission during the first 30 days after diagnosis.
• Mortality at 30 days after the diagnosis and new AP episode during the 30-day follow-up.
• EuroQoL in the outpatient clinic visit.
• Adverse Events

The BISAP score is a prognostic scoring system for early identification of patients at risk for in-hospital mortality.^[13]^ The BISAP uses 5 points: Blood Urea Nitrogen (BUN) > 25 mg/dL, impaired mental status, systemic inflammatory response syndrome,^[11]^ age > 60 years, and pleural effusions. BISAP scores ≥ 3 have been associated with higher mortality.^[13]^

The Charlson Comorbidity Index consists of 19 items displaying different clinical weights on the basis of the adjusted risk of one-year mortality, controlling for severity of illness and age. It has been shown to accurately relate with the 10-year life expectancy.^[14]^ The total score consists in a simple sum of the weights, with higher scores indicating not only a greater mortality risk but also more severe comorbid conditions.^[14]^

The pain VAS is a self-reported scale consisting of a horizontal or vertical line, usually 10 centimeters long (100 mm) anchored at the extremes by 2 verbal descriptors referring to the pain status. An introductory question (with or without a time recall period) asks the patient to tick the line on the point that best refers to his/her pain.^[15]^

The EuroQoL questionnaire is a widely used instrument to measure health-related QoL. We will use the EQ-5D-3L version, which essentially consists of 2 pages: the EQ-5D descriptive system and the EQ-5D visual analogue scale (EQ-VAS). The EQ-5D-3L descriptive system comprises 5 dimensions: mobility, self-care, usual activities, pain/discomfort, and anxiety/depression. Each dimension has 3 levels: no problems, some problems, extreme problems (labeled 1–3). The EQ-VAS records the respondent’s self-rated health on a vertical VAS where the endpoints are labeled “The best health you can imagine” and “The worst health you can imagine.”^[16]^

### 2.11. Plans to promote participant retention and complete follow-up

Follow-up will be performed either at homecare or during hospital admission. Notably, patients will be required to attend to the outpatient clinic 5 to 7 days after being discharged from the emergency department (as a safety measure); however, we do not think this will imply a considerable extra-effort that would prevent patients to complete follow-up. In each medical follow-up visit (be it face-to-face or by phone), the clinical investigator in charge will remind the patients of the importance of correctly following the study procedures and encourage them to carry on in the clinical trial.

Protocol deviations will be documented and explained in detail by the study team. In the event of a “serious” protocol violation, the monitoring team will record all protocol breaches/deviations. The sponsor will review all protocol deviations and assess whether they represent a “serious” violation according to Good Clinical Practice guidelines. The sponsor will inform the IRB of any protocol breach/deviation that could impact on patient safety and on data integrity.

### 2.12. Data management

An electronic case report form (eCRF), based on REDCap platform (REDCap Consortium), will be created *ad hoc* for this study in coordination with the Biostatistics Unit of the IDIBELL. It does not collect data that allows patient identification.

Before closing the database for analysis, the data manager and the principal investigator will check the completeness and accuracy of the recorded data.

### 2.13. Statistical methods

Considering this is a pilot study, analyses will be primarily performed in the per protocol population; however, we will also perform intention-to-treat analyses whenever feasible. We will perform a general descriptive analysis of all study variables. The results will be expressed as means (standard deviation [SD]) or medians (range) for the quantitative variables and as absolute and relative frequencies for the categorical variables.

We will use Pearson’s χ^2^ (or Fisher’s exact test, when appropriate) to perform a comparative analysis of the categorical variables. For the continuous variables, we will use the Student’s *t* test (if the distribution is normal) or the Mann–Whitney *U* for the non-parametric variables. Unless stated otherwise, two-sided 95% Confidence Intervals will be reported.

### 2.14. Data monitoring, description of any interim analyses and stopping guideline

A DSMC will be created *ad hoc* and will be composed by a specialist in Clinical Pharmacology and Clinical Trial Methodology (Dr Sebastián Videla), a specialist in Clinical Pharmacology and Pharmacovigilance (Dr Dolores Rodríguez) and by a specialist surgeon (Dr Juan Fabregat). None of its members will be involved with patient evaluation or treatment administration. The aim of this DSMC is to evaluate the safety of this clinical trial. It will meet twice, when half the calculated sample size has been included (i.e., 154 patients) and when the last patient has been included. The clinical trial could be halted if the DSMC deems necessary.

As mentioned before, an interim analysis (for safety purposes) will be performed when half the estimated sample size is included.

### 2.15. Harms

Adverse events recorded during the study will be coded according to the latest available version of the MedDRA dictionary and will be described using absolute and relative frequencies by study group, according to severity and its causal relation with treatment.

Serious adverse events will be described by study group and the 95% CI of the difference between both groups will be calculated.

### 2.16. Auditing

The Investigator shall allow direct access to trial data and documents for monitoring, audits and/or inspections by competent regulatory or health authorities. As such, eCRFs, source records and other trial-related documentation must be kept current, complete, and accurate at all times.

### 2.17. Ethics and dissemination

The study protocol (Version 8 – March 2, 2023) was approved by the local Institutional Review Board of the Bellvitge University Hospital (Barcelona, Spain) (Ethics and Clinical Investigation Committee, code PR129/22, on March 9, 2023).

This Trial will be conducted according to the criteria set by the Declaration of Helsinki (revised on WMA 64th General Assembly, Fortaleza, Brazil, October 2013), Good Clinical Practice standards and applicable regulations. Every patient that accepts to participate will be requested to sign a written informed consent prior to initiating any research activities.

Furthermore, patients must be informed that their participation in this research is entirely voluntary, and that they can withdraw at any time, under no penalty risk whatsoever. Investigators’ participation in this study is free, voluntary, unpaid, and independent.

The level of confidentiality protection, in terms of personal data protection, as required by Spanish Law (Organic Law on Data Protection 3/2018), was also ensured. Every patient that accepts to participate in the study will be assigned consecutive numbers as they are enrolled, and these numbers (or codes) will be used in the eCRF, instead of personal data. The data collected will be encoded, so that the patient to whom they correspond is not identified.

### 2.18. Plans for communicating important protocol amendments to relevant parties

Major protocol changes will be submitted for IRB approval, and minor changes will be informed to the IRB. As per good clinical practice, trial participants will be informed of any significant changes during the trial.

### 2.19. Who will obtain informed consent?

Study team members from the Digestive and General Surgery Department and the Gastroenterology Department will inform the screened patients about the study and ask them to sign the written informed consent form at Time 2. If he/she is interested to participate, the investigator of the study team will double-check the eligibility criteria before obtaining the signed written informed consent.

### 2.20. Confidentiality

The results from this clinical trial are confidential and may not be transferred to third parties in any form or manner without written permission from the Sponsor. All individuals involved in the clinical trial are bound to this confidentiality clause in line with the Regulation (EU) 2016/679 of the European Parliament and of the Council of April 27, 2016 on the protection of natural persons with regard to the processing of personal data and on the free movement of such data, as well as all other valid and applicable laws and regulations, such as the “*Ley Orgánica 3/2018, de 5 de diciembre, de Protección de Datos Personales y garantía de los derechos digitales*” [Personal Data Protection and Digital Rights Assurance Law]. Therefore, patient data will be pseudonymized.

While obtaining a signature for the Written Informed Consent, the Investigator will request written permission from the patient to directly access his/her data. With this permission granted, the patient’s data may be examined, analyzed, verified, and reproduced for the evaluation of the clinical trial.

Data will be anonymized, so that the corresponding patient cannot be identified. Patient data will also be dissociated. Patients will be assigned consecutive numbers as they are enrolled in the study, and these identification numbers (or codes) will be used in the eCRF; the full name of the patient will not be included in the eCRFs. The principal investigator of each center will keep an updated patient identification list containing the name, clinical history number and the patient’s identification number (or code) for the clinical trial.

The study monitor may have access to the patient’s identity and data related to the study monitoring procedures. Any person with direct access to the data (Regulatory Authorities, Trial Monitors, and Auditors) will take all possible precautions to maintain the confidentiality of patient’s identities. It is the Investigator’s responsibility to obtain a written informed consent from the study patients. It is the Trial Monitor’s responsibility to make sure that each patient has given his/her written consent to allow this direct access.

The Investigator shall ensure that the documents provided to the Sponsor do not contain the patient’s name or any identifiable data.

### 2.21. Declaration of interests

The authors have no conflict of interests to disclose.

### 2.22. Availability of data and materials

The database management and statistical analysis will be carried out by the Biostatistics Unit of the IDIBELL. Only the sponsor and the biostatisticians will have access to the final trial dataset. The datasets used and/or analyzed during the study will be available from the corresponding author upon reasonable request.

### 2.23. Ancillary and post-trial care

A specific insurance has been hired *ad hoc*, in case of any harm related to a patient’s participation.

### 2.24. Dissemination policy: trial results, authorship

The study findings will be submitted to a peer-reviewed journal for publication and presented at relevant national and international scientific meetings. The authorship is based on the criteria set by the International Committee of Medical Journal Editors.

### 2.25. Plans to give access to the full protocol, participant level-data and statistical code

The study protocol is registered on clinicaltrials.gov (NCT05473260). The Sponsor may grant access to the full protocol (in Spanish) on a case-by-case basis and upon reasonable request by the interested party.

No public access to the patient dataset is planned to be given at this moment. Professor Judit Peñafiel, the Head of the Biostatistics Unit, will oversee the dataset and granting access to this information will be evaluated on a case-by-case basis and upon reasonable request by the interested party.

## 3. Discussion

Acute pancreatitis is a high-incidence disease and current guidelines support in-hospital care, despite the severity. This implies a high economic burden in healthcare systems worldwide. Nonetheless, recent evidence suggests that mild acute pancreatitis can be safely and effectively treated with home monitoring with regular visits by a nurse under the supervision of a physician.^[4]^ This approach may produce considerable cost savings and positively impact patients’ quality of life.

This study may have limitations due to the open-label design and patient-reported outcomes. A patient’s knowledge of the assigned treatment arm could influence their view and reporting of their symptoms, although we do not believe this will be the case since treatments will be very similar, except for the administration route (for analgesic medication) and for the type of oral feeding. Additionally, following the usual clinical practice when managing admitted patients could generate different approaches between the different participating medical centers; however, the acute pancreatitis approach is quite standardized among the participating hospitals, with minor differences in the medication brands used by each center (similar to what is found in the real world).

We expect the RHINO trial results to show that home monitoring is effective, safe, and not inferior to hospitalization for managing mild acute pancreatitis. We also expect to show that the economic costs are lower when treating these patients with home monitoring. Altogether, this multicenter large sample-sized trial could change the standard clinical approach to mild acute pancreatitis in Spain, kickstart similar trials throughout the world, optimize the use of limited healthcare budgets, and improve patients’ overall quality of life.

## Acknowledgments

We would like to thank the Spanish Gastroenterology Association (AEG) for the grant awarded. We also would like to thank the *IDIBELL Foundation*, the *CERCA Program/Generalitat de Catalunya*, and the *Departament de Ciències Clíniques, Facultat de Medicina i Ciències de la Salut, Universitat de Barcelona (UB*) for the institutional support provided.

We would like to thank all RHINO study group members:

•Dr Marta Gil Barrionuevo, Hospital de Viladecans;

•Dr Marta Arnau Vidal, Hospital de Viladecans;

•Dr Francisco Garcia Borobia, Corporació Sanitaria Parc Taulí;

•Dr Anna Muñoz Campaña, Corporació Sanitaria Parc Taulí;

•Dr Sergio González Martínez, Hospital Sant Joan Despí Moisés Broggi - Consorci Sanitari Integral (CSI);

•Dr Andrea Sanz Llorente, Hospital Sant Joan Despí Moisés Broggi - Consorci Sanitari Integral (CSI);

•Dr Stephani Tasayco Huamán, Hospital Universitari Vall Hebrón;

•Dr Elisabeth Pando Rau, Hospital Universitari Vall Hebrón;

•Dr Andrea Álvarez Torrado, Hospital de Igualada - Consorci Sanitari de l’Anoia (CSA);

•Dr Elisabet Baena Sanfeliu, Hospital de Igualada - Consorci Sanitari de l’Anoia (CSA);

•Dr Catalina Uribe Galeano, Hospital Fundació Sant Joan de Déu de Martorell;

•Dr Ione Fornaguera Marimon, Hospital Fundació Sant Joan de Déu de Martorell;

•Dr Gian Pier Protti Ruíz, Hospital Germans Trias i Pujol de Badalona;

•Dr Alba Zarate Pinedo, Hospital Germans Trias i Pujol de Badalona;

•Dr Anna Arroyo Serrano, Hospital de Terrassa;

•Dr Andrea Adroher Alfonso, Hospital de Terrassa;

•Dr Santiago Sánchez Cabús, Hospital de la Santa Creu i Sant Pau;

•Dr Rodrigo Medrano, Hospital de la Santa Creu i Sant Pau.

## Author contributions

**Conceptualization:** Maria Sorribas, Juli Busquets.

Funding acquisition: Juli Busquets.

Investigation: Maria Sorribas, Núria Peláez, Luis Secanella, Silvia Salord, Sònia Sarret, Juli Busquets.

Methodology: Thiago Carnaval, Sebastián Videla, Juli Busquets.

Writing – original draft: Maria Sorribas, Thiago Carnaval, Sebastián Videla, Juli Busquets.

Writing – review & editing: Maria Sorribas, Thiago Carnaval, Sebastián Videla, Juli Busquets.

## Supplementary Material

## References

[1] Mederos MA Reber HA Girgis MD. Acute pancreatitis: a review. JAMA. 2021;325:382–90. 33496779 10.1001/jama.2020.20317

[2] Lankisch PG Apte M Banks PA. Acute pancreatitis. Lancet. 2015;386:85–96. 25616312 10.1016/S0140-6736(14)60649-8

[3] Valverde-Lopez F Wilcox CM Redondo-Cerezo E. Evaluation and management of acute pancreatitis in Spain. Gastroenterol Hepatol. 2018;41:618–28. 30149943 10.1016/j.gastrohep.2018.06.012

[4] Ince AT Senturk H Singh VK. A randomized controlled trial of home monitoring versus hospitalization for mild non-alcoholic acute interstitial pancreatitis: a pilot study. Pancreatology. 2014;14:174–8. 24854612 10.1016/j.pan.2014.02.007

[5] Yao Q Liu P Peng S. Effects of immediate or early oral feeding on acute pancreatitis: a systematic review and meta-analysis. Pancreatology. 2022;22:175–84. 34876385 10.1016/j.pan.2021.11.009

[6] Working Group IAP/APA Acute Pancreatitis Guidelines. IAP/APA evidence-based guidelines for the management of acute pancreatitis. Pancreatology. 2013;13(4 Suppl 2):e1–15. 24054878 10.1016/j.pan.2013.07.063

[7] Eckerwall GE Tingstedt BB Bergenzaun PE. Immediate oral feeding in patients with mild acute pancreatitis is safe and may accelerate recovery--a randomized clinical study. Clin Nutr. 2007;26:758–63. 17719703 10.1016/j.clnu.2007.04.007

[8] Guo QH Tian XY Qin YL. Immediate enteral nutrition can accelerate recovery and be safe in mild acute pancreatitis: a meta-analysis of randomized controlled trials. Heliyon. 2022;8:e08852. 35198753 10.1016/j.heliyon.2022.e08852 PMC8844690

[9] Zhang J Zhu S Tan D. A meta-analysis of early oral refeeding and quickly increased diet for patients with mild acute pancreatitis. Saudi J Gastroenterol. 2019;25:14–9. 30226482 10.4103/sjg.SJG_240_18 PMC6373213

[10] Banks PA Bollen TL Dervenis C.; Acute Pancreatitis Classification Working Group. Classification of acute pancreatitis--2012: revision of the Atlanta classification and definitions by international consensus. Gut. 2013;62:102–11. 23100216 10.1136/gutjnl-2012-302779

[11] Singer M Deutschman CS Seymour CW. The Third International Consensus Definitions for Sepsis and Septic Shock (Sepsis-3). JAMA. 2016;315:801–10. 26903338 10.1001/jama.2016.0287 PMC4968574

[12] Serra Pla S Garcia Monforte N Garcia Borobia FJ. Early discharge in mild acute pancreatitis. is it possible? observational prospective study in a tertiary-level hospital. Pancreatology. 2017;17:669–74. 28851510 10.1016/j.pan.2017.07.193

[13] Papachristou GI Muddana V Yadav D. Comparison of BISAP, Ranson’s, APACHE-II, and CTSI scores in predicting organ failure, complications, and mortality in acute pancreatitis. Am J Gastroenterol. 2010;105:435–41; quiz 442. 19861954 10.1038/ajg.2009.622

[14] Charlson ME Carrozzino D Guidi J. Charlson comorbidity index: a critical review of clinimetric properties. Psychother Psychosom. 2022;91:8–35. 34991091 10.1159/000521288

[15] Chiarotto A Maxwell LJ Ostelo RW. Measurement properties of visual analogue scale, numeric rating scale, and pain severity subscale of the brief pain inventory in patients with low back pain: a systematic review. J Pain. 2019;20:245–63. 30099210 10.1016/j.jpain.2018.07.009

[16] EuroQol Group. EuroQol – a new facility for the measurement of health-related quality of life. Health Policy. 1990;16:199–208. 10109801 10.1016/0168-8510(90)90421-9

