# Supplementary material for: Home monitoring vs hospitalization for mild acute pancreatitis. A pilot randomized controlled clinical trials
Source: Medicine (Baltimore). 2023 May 17;102(20):e33853. doi: 10.1097/MD.0000000000033853 (PMC10194650; doi:10.1097/MD.0000000000033853)
Supplement: Supplementary file 1 [file medi-102-e33853-s001.pdf]

Supplementary Material 1

Written Informed Consent

CONSENTIMIENTO INFORMADO **ESTUDIO RHINO**

ENSAYO CLÍNICO PILOTO ALEATORIZADO SOBRE ALTA PRECOZ FRENTE INGRESO HOSPITALARIO EN PACIENTES CON PANCREATITIS AGUDA LEVE

Yo (nombre y apellidos en mayúsculas) ____:

He leído y comprendido la información que se me ha entregado.	Sí ☐	No ☐
He recibido suficiente información del estudio y he podido realizar las preguntas necesarias	Sí ☐	No ☐
He entendido que soy libre de retirarme del estudio en cualquier momento sin dar ningún motivo y sin que influya en mi tratamiento	Sí ☐	No ☐
Acepto participar en el estudio voluntariamente	Sí ☐	No ☐
Autorizo que los remanentes del material biológico y sus datos asociados sean gestionados en régimen de biobanco, una vez finalizado el estudio.	Sí ☐	No ☐

Autorizo mi inclusión en el estudio.

Firma del paciente: **Firma del investigador:**

--- ---

Fecha y lugar:

INFORMED CONSENT **RHINO TRIAL**

HOME MONITORING VS. HOSPITALIZATION FOR MILD ACUTE PANCREATITIS. A PILOT RANDOMIZED CONTROLLED CLINICAL TRIAL (RHINO TRIAL)

I (given name and surname in capital letters) ____:

Have read and understood the contents that were given me.	Yes ☐	No ☐
Have received enough information regarding the study and been able to ask all the questions I deemed necessary.	Yes ☐	No ☐
I have understood that I am free to drop out from the study whenever I want and without needing to provide any justification and that such action shall not influence any healthcare treatment I receive thereafter.	Yes ☐	No ☐
Voluntarily accept to participate in this trial.	Yes ☐	No ☐
Authorize the remnants of my biological material and its associated data to be managed in a biobank once the study ends.	Yes ☐	No ☐

I accept to be included in this trial.

Patient signature: **Investigator signature:**

-- --

Date and location:
