# Supplementary material for: Home monitoring vs hospitalization for mild acute pancreatitis. A pilot randomized controlled clinical trials
Source: Medicine (Baltimore). 2023 May 17;102(20):e33853. doi: 10.1097/MD.0000000000033853 (PMC10194650; doi:10.1097/MD.0000000000033853)
Supplement: Supplementary file 2 [file medi-102-e33853-s002.pdf]

Supplementary Material 2

Patient Information Sheet

ENSAYO CLÍNICO PILOTO ALEATORIZADO SOBRE ALTA PRECOZ FRENTE INGRESO HOSPITALARIO EN PACIENTES CON PANCREATITIS AGUDA LEVE.

La pancreatitis aguda es la inflamación de la totalidad o parte del páncreas. Las causas principales de la inflamación de dicho órgano son los cálculos biliares, el alcohol y ciertos medicamentos. El 80% de las pancreatitis agudas son de tipo leve, y solo requieren de un tratamiento de soporte para el control del dolor, las náuseas y una adecuada reposición de líquidos.

Actualmente, todos los pacientes diagnosticados de una pancreatitis aguda leve son ingresados en una unidad de hospitalización convencional a cargo del servicio de Cirugía General y Digestiva o Digestología, donde son controlados, se administra tratamiento sintomático y se realiza una ecografía abdominal para valorar cual es la causa de la inflamación del páncreas. Un 20% de las pancreatitis agudas presentan criterios de gravedad desde su inicio, y requieren de un tratamiento agresivo con ingreso en una unidad de cuidados intensivos.

Este documento tiene como objetivo informarle y pedirle su consentimiento para ser incluido en un estudio cuyo objetivo es demostrar que el alta precoz (inferior a 24h) en pacientes con pancreatitis aguda leve es factible y segura.

El objetivo del presente estudio es comparar dos tipos diferentes de manejo de la pancreatitis aguda leve de origen no alcohólico: El ingreso hospitalario y el manejo ambulatorio.

En caso de que usted acepte participar en este estudio, una vez se diagnostique en urgencias de una pancreatitis aguda leve de origen no alcohólico, se decidirá de forma aleatoria, es decir debida al azar, si se procede al ingreso hospitalario o si se opta por un control ambulatorio.

Estos procedimientos no van a interferir en la normal asistencia al paciente y no le representarán molestias ni riesgos adicionales. Usted no obtendrá ningún beneficio inmediato de la participación en este estudio. Sin embargo, la información obtenida nos permitirá realizar con evidencia científica un mejor manejo terapéutico con una mayor calidad de vida de los pacientes que presenten en un futuro una pancreatitis aguda leve de origen no alcohólico.

Los datos serán codificados mediante un sistema numérico y tratados de forma confidencial, de forma que solo los investigadores y los miembros del comité ético tendrán acceso a sus datos personales, que no constaran en la base de datos. Además, según la Ley Oficial de Protección de Datos, usted tendrá acceso a sus datos, con derecho a obtener, rectificar y cancelar los mismos.

Su participación en este estudio es completamente voluntaria. Usted es libre de retirarse del estudio en cualquier momento si así lo cree conveniente, sin tener que especificar los motivos, y sin que su decisión perjudique en el futuro la atención médica que merece.

Toda la información resultante de su participación en el estudio será almacenada y analizada en un ordenador y se tratará de forma confidencial según la legislación vigente. Los resultados de este estudio pueden ser publicados en revistas médicas. Sin embargo, su nombre no aparecerá en ningún documento.

Este estudio ha sido aprobado por el Comité Ético de Investigación Clínica CEIC del Hospital Universitari de Bellvitge número **PR129/22** y está cubierto por una póliza de seguro específica con el fin de cubrir la Responsabilidad Civil derivada de la realización de Ensayos Clínicos.

HOME MONITORING VS. HOSPITALIZATION FOR MILD ACUTE PANCREATITIS. A PILOT RANDOMIZED CONTROLLED CLINICAL TRIAL (RHINO TRIAL)

Acute pancreatitis is a complete or partial inflammation of the pancreas. The main causes are gallstones, alcohol, and certain medications.

Approximately 80% of all cases of acute pancreatitis are mild and only require supportive care for pain and nausea control and an adequate fluid replacement. Currently, all patients in our setting diagnosed with mild acute pancreatitis are admitted to a conventional hospitalization ward under the supervision of the Digestive and General Surgery Department or the Gastroenterology Department. Symptomatic treatment is administered, and abdominal ultrasound is performed to assess the cause of pancreatic inflammation. The remaining 20% fulfill the severity criteria from its onset onwards and require intensive care support.

The purpose of this information sheet is to explain the disease background and ask for your consent to be included in a study whose objective is to demonstrate that discharging patients with mild acute pancreatitis early (in less than 24h) is feasible and safe.

The present study will compare two different approaches to mild non-alcoholic acute pancreatitis: hospital admission and outpatient management.

Should you agree to participate in this study, once mild non-alcoholic acute pancreatitis is diagnosed in the emergency room, you will be randomly assigned (by chance) to either hospital admission or outpatient management.

These procedures will not interfere with standard patient care and will not represent any discomfort or additional risks. You will not get any immediate benefit from taking part in this study. However, the information obtained will allow us to provide, with scientific evidence, better therapeutic management and a higher quality of life for mild non-alcoholic acute pancreatitis patients in the future.

The data will be coded using a numerical system and treated confidentially so that only the researchers and members of the ethics committee will have access to your personal data, which will not appear in the database. Additionally, per the Official Data Protection Law, you will have access to your personal data at any time and have the right to obtain and rectify it, as well as to withdraw authorization whenever you deem necessary.

Your participation in this study is completely voluntary. You are free to withdraw from the study at any time if you deem it convenient, without specifying the reasons, and without your decision affecting any future medical care provided.

All information resulting from your participation in the study will be digitally stored and analyzed, and treated confidentially under the current legislation. The results of this study may be published in medical journals. However, your name or any data that could allow your identification will not appear on any documents.

This study has been approved by the Clinical Research Ethics Committee CEIC of the Bellvitge University Hospital number **PR129/22** and a specific insurance has been hired to cover potential Civil Liability issues derived from the performance of Clinical Trials.
